# Pre-pandemic diabetes and risk of long COVID: longitudinal evidence

**DOI:** 10.1007/s40200-025-01731-4

**Published:** 2025-09-19

**Authors:** Yusuff Adebayo Adebisi, Anas Ali Alhur, Najim Z. Alshahrani, Victor C. Cañezo, Edgar G. Cue, Don Eliseo Lucero-Prisno

**Affiliations:** 1 College of Social Sciences, University of Glasgow, 40 Bute Gardens, Glasgow, G12 8RT United Kingdom; 2 College of Public Health and Health Informatics, University of Hail, Hail, Saudi Arabia; 3 College of Public Health, Imam Abdulrahman Bin Faisal University, Dammam, Saudi Arabia; 4 Department of Family and Community Medicine, Faculty of Medicine, University of Jeddah, Jeddah, Saudi Arabia; 5 Office of the University President, Biliran Province State University, Naval, Leyte Philippines; 6 Office of the University President, Mountain Province State University, Bontoc, Mountain Province Philippines; 7 Department of Global Health and Development, London School of Hygiene and Tropical Medicine, London, United Kingdom; 8 Center for University Research, University of Makati, Makati City, Philippines; 9 Research Office, Palompon Institute of Technology, Palompon Institute of Technology, Palompon, Leyte Philippines

**Keywords:** Long COVID, Diabetes, Post-COVID syndrome, Chronic illness, Cohort study

## Abstract

**Objective:**

To examine whether pre-pandemic diabetes is associated with an increased risk of Long COVID in a nationally representative UK cohort.

**Methods:**

We conducted a prospective cohort analysis using data from the UK Household Longitudinal Study. A total of 11,669 adults aged ≥ 16 years were followed from Wave 10 (2018–19) to Wave 14 (2022–23). The primary exposure, pre-pandemic diabetes, was defined at baseline (Wave 10) based on self-report of a doctor diagnosis. The primary outcome, Long COVID, was assessed at follow-up (Wave 14) and defined as self-reported symptoms lasting more than 12 weeks after a COVID-19 infection that could not be explained by another cause. Modified Poisson regression models with robust standard errors were used to estimate relative risks of Long COVID associated with pre-pandemic diabetes. Predictive margins were then calculated to obtain adjusted probabilities.

**Results:**

At follow-up, 1,076 participants (9.2%) reported Long COVID. In the unadjusted model, participants with pre-pandemic diabetes had a 36% higher risk of Long COVID compared with those without diabetes (RR = 1.36, 95% CI: 1.09–1.69, *p* = 0.006). After adjusting for age and sex, the relative risk increased to 1.43 (95% CI: 1.15–1.79, *p* = 0.002). In the fully adjusted model, which controlled for age, sex, ethnicity, education, income satisfaction, smoking, and other long-standing illness, the relative risk of Long COVID in participants with diabetes was 1.60 (95% CI: 1.27–2.02, *p* < 0.001). The adjusted predicted probability of long COVID was 14.4% (95% CI: 11.2–17.6) among those with diabetes, compared with 9.0% (95% CI: 8.5–9.5) among those without.

**Conclusions:**

In this nationally representative prospective cohort, pre-pandemic diabetes emerged as an independent risk factor for Long COVID. Enhanced surveillance and targeted support for individuals with diabetes may be warranted in Long COVID care strategies.

## Introduction

The long-term health consequences of COVID-19 have emerged as a major public health concern [1, 2]. A significant proportion of individuals infected with SARS-CoV-2 go on to experience persistent symptoms lasting weeks or months after the acute phase of illness, a condition widely referred to as Long COVID [3, 4]. Symptoms range from fatigue and breathlessness to cognitive impairment, musculoskeletal pain, and autonomic dysfunction, with varying degrees of severity and impact on daily life [3]. The UK Office for National Statistics estimates that over one million people in the UK experience symptoms consistent with Long COVID [5], underlining its growing clinical and societal burden.

Emerging research has identified several factors that may increase the risk of developing Long COVID, including female sex, older age, socioeconomic disadvantage, and pre-existing health conditions [6–9]. Among these, diabetes has received particular attention due to its well-established role in impairing immune function, promoting systemic inflammation, and increasing vulnerability to infections [10–13]. While diabetes is known to increase the severity of acute COVID-19 outcomes, its role in shaping long-term recovery trajectories remains less well understood. Existing studies have largely relied on hospital-based or convenience samples, with limited generalisability to the broader population [13–15]. Moreover, few have used prospectively measured pre-pandemic diabetes status to establish temporal directionality [14].

In this study, we used data from a nationally representative UK longitudinal survey to assess whether individuals with pre-pandemic diabetes were at higher risk of developing Long COVID following a confirmed COVID-19 infection.

## Methods

### Study design, data source, and study population

This was a prospective cohort analysis using data from the UK Household Longitudinal Study (UKHLS), also known as Understanding Society. The survey is a nationally representative panel study that follows individuals aged 16 years and older across the United Kingdom. It collects annual data on health, education, employment, and behaviours using a complex stratified clustered sampling design, and includes ethnic minority boost samples to improve representativeness [16, 17].

For this study, Wave 10 (2018–19) served as the pre-pandemic baseline and Wave 14 (2022–23) as the follow-up period during which Long COVID outcomes were assessed. Wave 10 included 34,319 adult respondents, and Wave 14 included 35,471. Of the 46,405 unique individuals who participated in either or both waves, 23,385 (50.4%) were successfully matched across Waves 10 and 14 using a unique personal identifier (pidp). Among these matched respondents, 11,695 adults reported a positive COVID-19 test result in Wave 14. After excluding 26 individuals who were interviewed by proxy or who provided “don’t know” or “refused” responses to the Long COVID question, the final analytic sample comprised 11,669 adults with confirmed COVID-19 and valid Long COVID outcome data. All respondents in this analytic sample also had complete data on pre-pandemic diabetes status, ensuring no loss of sample due to missing exposure information.

### Exposure: Pre-pandemic diabetes

The primary exposure was pre-pandemic diabetes, assessed in Wave 10 (2018–19) of the UKHLS, prior to the onset of the COVID-19 pandemic. Participants were asked whether a doctor or other health professional had ever told them they had diabetes. Responses were used to construct a binary indicator variable, coded 1 for individuals who reported having either type 1 or type 2 diabetes, and 0 for those who reported no diabetes diagnosis. This self-reported, doctor-diagnosed measure is commonly used in population-based cohort studies and has demonstrated acceptable validity for identifying diabetes cases in UK survey data [18–20]. The exposure variable thus captures long-standing, clinically recognised diabetes status prior to any COVID-19 infection or related outcomes. Type 1 and type 2 diabetes were combined into a single category to maximise statistical power.

### Outcome: long COVID

The primary outcome was self-reported Long COVID, measured in Wave 14 (2022–23) of the UKHLS. Respondents who reported testing positive for COVID-19 were subsequently asked whether they had experienced symptoms that persisted for more than 12 weeks and could not be explained by something else. Based on responses to this question, a binary outcome variable was created: individuals were coded 1 if they reported experiencing Long COVID symptoms, and 0 if they did not. Respondents who were interviewed by proxy or who answered “don’t know” or “refused” were excluded from the analytic sample.

### Covariates

Covariates were selected a priori based on existing literature on the social and health determinants of Long COVID [21–23]. All covariates were measured at baseline in Wave 10 (2018–19), prior to the onset of the COVID-19 pandemic. Age was grouped into seven categories (16–29, 30–39, 40–49, 50–59, 60–69, 70–79, and 80 + years). Sex was coded as male or female. Ethnicity was categorised into five groups: White, Mixed, Asian, Black, and Other. Educational attainment was based on the highest qualification achieved and grouped into four categories: no qualifications, lower secondary, upper secondary, and tertiary education, with a separate category for missing responses. Household income satisfaction was assessed via self-report and grouped into four categories: dissatisfied, neutral, satisfied, and missing. Smoking status was included as a binary variable (current smoker vs. non-smoker). Finally, to distinguish the independent effect of diabetes, we adjusted for the presence of any long-standing illness excluding diabetes, based on responses to a checklist of doctor-diagnosed conditions in Wave 10.

### Statistical analysis

We first described baseline characteristics of the study population by Long COVID status using Pearson chi-square tests for categorical variables. To estimate the association between pre-pandemic diabetes and the risk of developing Long COVID, we fitted a series of Poisson regression models with robust standard errors. This approach allows for the estimation of relative risks (RRs) in the context of a binary outcome and is appropriate when there is no evidence of overdispersion [24].

Modelling proceeded in a stepwise fashion. The initial model included diabetes status as the sole predictor (crude model). We then sequentially added groups of covariates to assess their influence on the association. The second model adjusted for age group and sex. The third model further included ethnicity, highest educational attainment, and satisfaction with household income as indicators of socioeconomic position. The fourth model added smoking status, and the fifth and final model additionally controlled for the presence of any long-standing illness other than diabetes. Multicollinearity was assessed using variance inflation factors (VIFs). All VIFs were well below the conventional threshold of 5, with a mean VIF of 1.82, indicating no significant collinearity between covariates. Goodness-of-fit was evaluated using the Pearson chi-square statistic, which showed no evidence of overdispersion (Pearson χ²(11,647) = 10,651.35, *p* = 1.000), supporting the use of Poisson regression with robust standard errors. To aid interpretation, we computed predictive margins from the final adjusted model to estimate the average predicted probability of long COVID for individuals with and without diabetes. These margins provide a useful summary of adjusted risks across the full sample.

To test the robustness of our findings, we conducted a sensitivity analysis restricted to participants who reported no long-standing illnesses other than diabetes, adjusting for the same covariates as in the main analysis. This approach enabled us to assess the association between diabetes and Long COVID in a comparatively “healthy” subpopulation, thereby reducing potential confounding by other chronic conditions.

All analyses were performed in Stata version 18.0 (StataCorp, College Station, TX, USA), using two-sided tests with a significance threshold of *p* < 0.05.

## Result

Table 1 presents the baseline characteristics of participants according to long COVID status. At follow up, 1,076 participants (9.2%) reported long COVID. The distribution of long COVID varied significantly across age groups (*p* < 0.001), with the highest prevalence observed among those aged 40–49 (23.1%) and 50–59 (25.3%). Participants aged 16–29 and those aged 70 years and older were least likely to report long COVID. Females were significantly more likely to report long COVID than males (63.4% vs. 36.6%, *p* = 0.002). There were no significant differences in long COVID prevalence by ethnicity (*p* = 0.534) or educational attainment (*p* = 0.540). However, income satisfaction showed a strong association with long COVID status (*p* < 0.001). Among those with long COVID, 30.6% reported being dissatisfied with their income compared with 21.3% of those without, while only 54.3% of participants with long COVID were satisfied with their income compared with 64.7% of those without. Smoking status demonstrated a borderline association with long COVID (*p* = 0.058), with current smokers slightly more likely to report long COVID than non-smokers (11.6% vs. 9.8%). Diabetes was significantly more common among participants with long COVID than among those without (7.0% vs. 5.1%, *p* = 0.007). Similarly, participants with long COVID were more likely to report having another long-standing illness besides diabetes (36.1% vs. 27.1%, *p* < 0.001).

**Table 1:** Baseline characteristics by long COVID status

Characteristic	No Long COVID (*n* = 10,593)	Long COVID (*n* = 1,076)	Total (*N* = 11,669)	*p*-value
Age group, *n* (%)				< 0.001
16–29	1,721 (16.2)	136 (12.6)	1,857 (15.9)	
30–39	1,771 (16.7)	189 (17.6)	1,960 (16.8)	
40–49	2,158 (20.4)	249 (23.1)	2,407 (20.6)	
50–59	2,187 (20.6)	272 (25.3)	2,459 (21.1)	
60–69	1,639 (15.5)	162 (15.1)	1,801 (15.4)	
70–79	946 (8.9)	60 (5.6)	1,006 (8.6)	
80+	171 (1.6)	8 (0.7)	179 (1.5)	
Sex, *n* (%)				0.002
Male	4,388 (41.4)	394 (36.6)	4,782 (41.0)	
Female	6,205 (58.6)	682 (63.4)	6,887 (59.0)	
Ethnicity, *n* (%)				0.534
White	9,169 (86.6)	919 (85.4)	10,088 (86.5)	
Mixed	180 (1.7)	25 (2.3)	205 (1.8)	
Asian	935 (8.8)	100 (9.3)	1,035 (8.9)	
Black	249 (2.4)	24 (2.2)	273 (2.3)	
Other	60 (0.6)	8 (0.7)	68 (0.6)	
Education level, *n* (%)				0.540
No qualification	564 (5.3)	53 (4.9)	617 (5.3)	
Lower secondary	2,553 (24.1)	275 (25.6)	2,828 (24.2)	
Upper secondary	2,286 (21.6)	244 (22.7)	2,530 (21.7)	
Tertiary	5,095 (48.1)	497 (46.2)	5,592 (47.9)	
Missing	95 (0.9)	7 (0.7)	102 (0.9)	
Income satisfaction, *n* (%)				< 0.001
Dissatisfied	2,256 (21.3)	329 (30.6)	2,585 (22.2)	
Neutral	1,260 (11.9)	140 (13.0)	1,400 (12.0)	
Satisfied	6,851 (64.7)	584 (54.3)	7,435 (63.7)	
Missing	226 (2.1)	23 (2.1)	249 (2.1)	
Smoking status, *n* (%)				0.058
No	9,555 (90.2)	951 (88.4)	10,506 (90.0)	
Yes	1,038 (9.8)	125 (11.6)	1,163 (10.0)	
Diabetes, *n* (%)				0.007
No diabetes	10,058 (95.0)	1,001 (93.0)	11,059 (94.8)	
Has diabetes	535 (5.1)	75 (7.0)	610 (5.2)	
Long-standing illness (excl. diabetes), *n* (%)				< 0.001
No	7,725 (72.9)	688 (63.9)	8,413 (72.1)	
Yes	2,868 (27.1)	388 (36.1)	3,256 (27.9)	

**Fig. 1:**
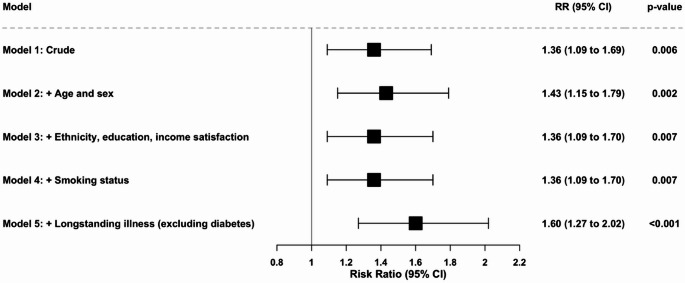
Crude and Adjusted Poisson regression models for the association between diabetes and long COVID. Models were sequentially adjusted as follows: Model 1 (crude, unadjusted); Model 2 (+ age and sex); Model 3 (+ ethnicity, education, and income satisfaction); Model 4 (+ smoking status); and Model 5 (+ longstanding illness, excluding diabetes). Risk ratios (RRs) and 95% confidence intervals (CIs) are shown. The vertical line indicates the null value (RR = 1)

Figure 1 shows the results of stepwise Poisson regression models estimating the relative risk of long COVID associated with pre-pandemic diabetes. In the unadjusted model (Model 1), individuals with diabetes had a 36% higher risk of reporting long COVID compared to those without diabetes (RR = 1.36, 95% CI: 1.09–1.69, *p* = 0.006). Adjustment for age and sex in Model 2 slightly strengthened the association (RR = 1.43, 95% CI: 1.15–1.79, *p* = 0.002). Adding ethnicity, educational attainment, and income satisfaction in Model 3 attenuated the estimate to an RR of 1.36 (95% CI: 1.09–1.70, *p* = 0.007), suggesting partial confounding by socioeconomic factors. Further adjustment for smoking status in Model 4 did not materially change the association (RR = 1.36, 95% CI: 1.09–1.70, *p* = 0.007). However, after controlling for other long-standing illnesses (excluding diabetes) in Model 5, the relative risk increased substantially (RR = 1.60, 95% CI: 1.27–2.02, *p* < 0.001), indicating that among individuals without additional chronic conditions, the independent association between diabetes and long COVID is stronger.

**Fig. 2:**
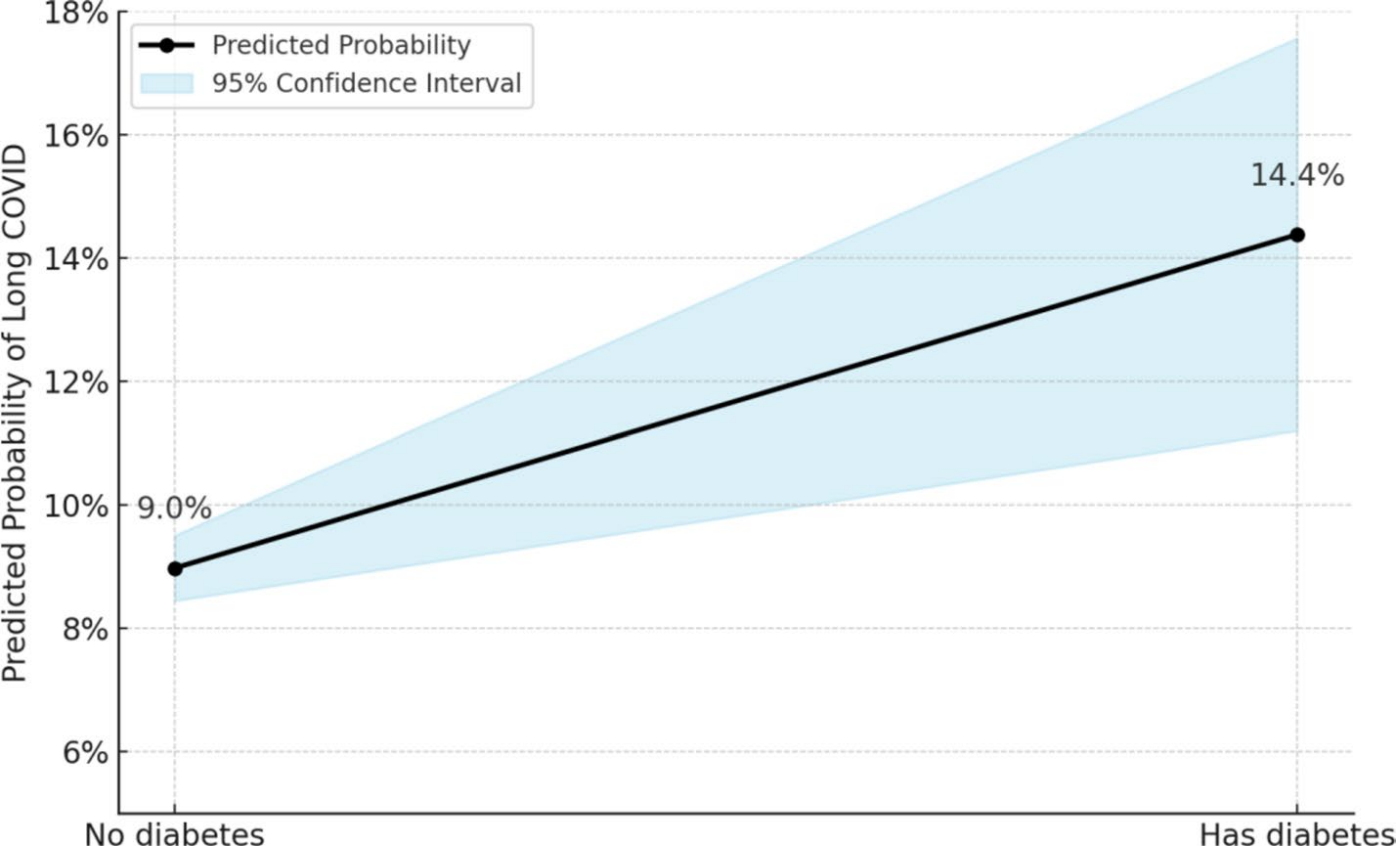
Adjusted predicted probability of long COVID by diabetes status. Predicted probabilities and 95% confidence intervals were derived from fully adjusted Poisson regression models controlling for age, sex, ethnicity, education, income satisfaction, smoking status, and long-standing illness (excluding diabetes)

Figure 2 presents the adjusted predicted probability of developing long COVID by diabetes status, derived from the fully adjusted Poisson regression model. Participants with diabetes had a significantly higher predicted probability of long COVID (14.4%, 95% CI: 11.2–17.6%) compared to those without diabetes (9.0%, 95% CI: 8.5–9.5%). This difference persisted after accounting for age group, sex, ethnicity, education, income satisfaction, smoking status, and the presence of other long-standing illnesses. The plot illustrates a clear elevation in risk associated with diabetes, supporting the regression findings and highlighting a meaningful disparity in long COVID outcomes by pre-pandemic diabetes status.

### Sensitivity analysis

To examine whether the association between diabetes and long COVID persisted in a healthier subpopulation, we conducted a sensitivity analysis restricted to participants who reported no long-standing illness other than diabetes (*n* = 8,413). Within this subgroup, the association between pre-pandemic diabetes and long COVID remained statistically significant and was slightly stronger than in the full sample. The adjusted relative risk was 1.58 (95% CI: 1.25–2.01, *p* < 0.001), indicating that even in the absence of other chronic conditions, individuals with diabetes were at substantially higher risk of developing long COVID compared to those without diabetes.

## Discussion

This study provides evidence that individuals with pre-pandemic diabetes were at substantially elevated risk of developing Long COVID after a confirmed COVID-19 infection. This association was robust to adjustment for key demographic and socioeconomic factors, smoking, and the presence of other long-standing illnesses. The fully adjusted model indicated a 60% higher risk of Long COVID among people with diabetes, with predicted probabilities of 14.4% for those with diabetes versus 9.0% for those without. Notably, this disparity persisted even after controlling for a wide range of potential confounders, suggesting that diabetes may act as an independent determinant of long-term COVID-19 sequelae. These findings are consistent with existing research from smaller clinical cohorts and registry studies that have identified diabetes as a risk factor for both acute COVID-19 severity and prolonged recovery [25–28], but our study is one of the first to demonstrate this relationship in a large, population-based sample using prospective, pre-pandemic baseline data.

The biological plausibility of the observed association is supported by several potential mechanistic pathways linking diabetes to prolonged post-viral illness. Diabetes is characterised by chronic low-grade inflammation, endothelial dysfunction, and immune dysregulation, all of which may impair viral clearance and exacerbate the systemic effects of SARS-CoV-2 [29]. Hyperglycaemia has been shown to facilitate viral replication and hinder macrophage and neutrophil function, while also altering cytokine profiles in ways that may predispose individuals to multisystem inflammation [30]. In addition, the ACE2 receptor, used by SARS-CoV-2 to enter host cells, is upregulated in people with diabetes, potentially increasing viral load and organ damage [31]. These pathophysiological processes may not only influence the severity of the initial infection but also contribute to the persistence of symptoms, including fatigue, breathlessness, cognitive dysfunction, and cardiovascular complications. Our finding that the association between diabetes and Long COVID was stronger in individuals without other long-standing illnesses reinforces the possibility that diabetes exerts an independent effect, rather than serving merely as a proxy for poor overall health or multimorbidity.

These findings have meaningful implications for public health planning, clinical practice, and patient care. First, they suggest that individuals with diabetes should be considered a high-risk group not only for acute COVID-19 outcomes but also for long-term post-viral complications. This has relevance for post-COVID care services, which should proactively monitor and support individuals with diabetes who have recovered from acute infection. Clinical guidelines could be updated to include routine screening for Long COVID symptoms among people with diabetes, especially given the substantial difference in predicted risk observed in this study. From a policy perspective, these findings underline the importance of integrated care pathways that bridge chronic disease management and post-infectious rehabilitation, particularly as Long COVID continues to place a burden on both patients and healthcare providers.

This study benefits from several methodological strengths. The use of a large, longitudinal, population-based cohort enhances generalisability to the adult UK population. The pre-pandemic measurement of diabetes ensures temporal ordering between exposure and outcome, reducing the risk of reverse causation. Nonetheless, this study is subject to limitations. Diabetes and Long COVID were self-reported, which may introduce misclassification bias. Individuals with a chronic condition such as diabetes may be more health-conscious, have more regular contact with healthcare services, and therefore be more likely to notice and report persistent symptoms, potentially leading to an overestimation of the observed association. Conversely, some participants may underrecognise or underreport symptoms, which could bias results towards the null. We were unable to distinguish between type 1 and type 2 diabetes or account for disease duration, severity, or treatment. Long COVID symptom data were collected retrospectively, which may have introduced recall bias. Additionally, as with all observational studies, unmeasured confounding remains a concern.

Despite these limitations, the results provide important insights into the long-term vulnerability of individuals with diabetes and a basis for targeted prevention and support strategies in the context of post-acute COVID-19 care.

## Conclusion

In this nationally representative prospective cohort study, we found that individuals with pre-pandemic diabetes were at significantly higher risk of developing Long COVID following a confirmed SARS-CoV-2 infection. The association remained robust after adjustment for demographic, socioeconomic, and health-related covariates and was stronger among individuals without additional long-standing illnesses, suggesting that diabetes may independently predispose individuals to prolonged post-COVID symptoms. These findings underline the need to prioritise people with diabetes in Long COVID screening, monitoring, and care efforts. They also highlight the importance of effective chronic disease management and glycaemic control not only for acute COVID-19 outcomes but also for mitigating long-term sequelae. As health systems grapple with the growing burden of Long COVID, targeted interventions for high-risk groups, including those with diabetes, will be essential to reducing disparities in recovery and improving long-term health outcomes.

## Data Availability

To download the dataset used in the analyses, please visit the [https://ukdataservice.ac.uk/find-data/browse/health/] (https://ukdataservice.ac.uk/find-data/browse/health)
